# Use of Dedicated Mobile Teams and Polio Volunteer Community Mobilizers to Increase Access to Zero-Dose Oral Poliovirus Vaccine and Routine Childhood Immunizations in Settlements at High Risk for Polio Transmission in Northern Nigeria

**DOI:** 10.1093/infdis/jiw520

**Published:** 2017-02-04

**Authors:** Kennedy M. Ongwae, Samuel B. Bawa, Faisal Shuaib, Fiona Braka, Melissa Corkum, Hammanyero K. Isa

**Affiliations:** 1 Health, United Nations Children's Fund (UNICEF), Islamabad, Pakistan; 2 Polio, World Health Organization; 3 Polio, National Primary Health Care Development Agency, Federal Ministry of Health, Abuja, Nigeria; 4 Polio, UNICEF, Kabul, Afghanistan

**Keywords:** personnel, resources, polio, eradication, routine, immunization, settlements

## Abstract

**Background:**

The Polio Eradication Initiative in Nigeria, which started >20 years ago, faced many challenges, including initial denial, resistance from communities, and prolonged regional safety concerns. These challenges led into the structuring of the response including the development of the National Emergency Action Plan, improved partner coordination and government engagement, and the establishment of a Polio Emergency Operations Centre. Although monthly supplementary immunization activities (SIAs) continued, the targeting of settlements at high risk for polio transmission with routine immunization (RI) and other selected primary healthcare (PHC) services using dedicated mobile teams and volunteer community mobilizers (VCMs) became a key strategy for interrupting polio transmission in the high-risk areas. These efforts could have contributed to the wild poliovirus–free 2-year period between 24 July 2014 and 11 August 2016, when 2 cases of the virus were reported from Borno State, Northern Nigeria.

**Methods:**

A narrative analysis of polio-related program and other official documents was conducted to identify the relevant human resources and their role in the Polio Eradication Initiative and in RI. The data used in the article was obtained from United Nations Children's Fund (UNICEF) and World Health Organization project reports and a draft evaluation report of the dedicated mobile teams approach in Northern Nigeria.

**Results:**

The data from 6 of the states that commenced the provision of polio, RI, and other selected PHC services using the dedicated mobile teams approach in 2014 showed an overall increase in the percentage of children aged 12–23 months in the settlements at high risk for polio transmission with a RI card seen, from 23% to 56%, and an overall increase in fully immunized children aged 12–23 months, from 19% to 55%. The number of newborns given the first dose of oral poliovirus vaccine (OPV) according to the RI schedule and the number of children given zero-dose OPV with the assistance of the VCMs similarly increased between 2013 and 2015. In 2015, VCMs helped track 167 092 newborns and also linked 156 537 infants aged <1 year to RI services in the 6 states.

**Conclusions:**

The analysis illustrates that polio personnel in Northern Nigeria are used in increasing access to zero-dose OPV, RI, and selected PHC services. The increase in the services generated represented the increasing role of the dedicated mobile teams and polio VCMs in strengthening RI.

The Polio Eradication Initiative (PEI) in Nigeria experienced major setbacks, starting in 2003 with the initial widespread boycott of poliovirus vaccinations in some of the country's Northern states [1–3]. This transitioned into poorly executed supplementary immunization activities (SIAs), a continuation of anti-poliovirus vaccine sentiments, and reduced access to services after the escalation of insecurity in Northern Nigeria in 2009 [4]. The SIAs have historically been used to perform mass immunization campaigns as a strategy for increasing coverage of childhood immunizations in Nigeria. The resistance, which continued till 2012, was deeply rooted in rumors about the safety of the poliovirus vaccine, compounded by strong religious, cultural, and political positions [1–3]. Starting in 2012, the country made significant gains in polio eradication, but the progress was set back when the country reported 2 new cases of wild poliovirus from Borno State in Northern Nigeria on 11 August 2016, slightly more than 2 years since the last case in 2014 [5].

During the period starting in 2012–2013, the country recorded continued polio transmission and a large number of polio cases in 11 high-risk states: Kaduna, Katsina, Niger, Jigawa, Zamfara, Kebbi, Sokoto, Borno, Bauchi, Kano, and Yobe [2]. After 2013, the number of cases decreased until 24 July 2014, when the last case of wild poliovirus in Nigeria was reported in Kano State. This led to the removal of Nigeria from the list of polio-endemic countries in 2015, ushering in a new phase of heightened surveillance and action [4].

In 2012, the high-level oversight over government and partners' efforts in polio eradication was consolidated through the establishment of a Presidential Task Force on Polio Eradication [6, 7]. The task force reactivated the Expert Review Committee on Polio Eradication in 2012. The task force also ensured that better and more focused and accountable planning and implementation processes were put in place and community actions and linkages were intensified to eradicate polio.

The PEI in Nigeria is supported by multiple planning processes, including the National Routine Immunization Strategic Plan 2013–2015, the Accountability Framework for Routine Immunization in Nigeria [8], and the Polio Endgame Strategic Plan 2013–2018 [9]. The objectives of the Polio Endgame Strategic Plan include polio detection and interruption, strengthening of routine immunization (RI) and the withdrawal of oral poliovirus vaccine (OPV), containment and certification of polio eradication, and polio legacy planning [9]. These strategies are further elaborated into yearly polio emergency plans [9]. The accountability framework is used to manage human and financial resources for the polio and RI program. As demonstrated through its application in Nigeria, the framework has the potential to improve staff performance and process indicators when used well [10].

Previous research on the impact of polio eradication activities on RI and primary healthcare (PHC) identified many missed opportunities [11]. To address some of the gaps, polio personnel in the form of dedicated mobile health teams and a network of volunteer community mobilizers (VCMs) are used to target settlements at high risk for polio transmission in Northern Nigeria. The equity-based intervention provide selected PHC services, including SIAs, to particularly hard-to-reach communities in the 12 states in Northern Nigeria with a high number of high-risk communities for polio transmission.

This article seeks to demonstrate and justify the use of the dedicated mobile teams and polio VCMs to improve not only polio immunization but also RI and other PHC services in the underserved communities thought to be at high risk for polio transmission in 6 of the 12 states. Our study is based on the initial 6 states (Bauchi, Borno, Kaduna, Kano, Katsina, and Yobe) that started implementation of the dedicated mobile teams and polio VCM initiative in 2014 and were included in the baseline assessment of 2014 as well as in the midterm assessment of 2015. The other 6 states started the implementation of the initiative in 2015 and hence have limited experience to learn from.

## METHODS

### Review of Program Reports and Official Documents

The identification of dedicated mobile teams and polio VCMs was established through a review of program reports and official documents on polio eradication in the country. A narrative analysis of the documentation was conducted to assess the use of dedicated mobile teams and polio VCMs in relation to polio eradication, RI as well as in the provision of other related PHC services.

### Review and Analysis of Data Generated From Services Offered by the Dedicated Mobile Teams and Polio VCMs

The dedicated mobile teams and polio VCMs provided polio eradication and RI related services. The data from the provision of these services was correlated and analyzed to generate trends according to the support provided to polio eradication and RI. In addition, baseline and midterm assessment of the services provided through the dedicated mobile teams and polio VCMs in the 6 Northern Nigeria states was used to assess the role of the polio personnel in increasing coverage for RI in the settlements at high risk for polio transmission. These settlements were preselected for intervention because of having a difficult terrain in combination with any of the following characteristics: locations of borders between wards, local government areas, or states; scattered households; inhabitation by nomadic population; location in a waterlogged or riverine area; difficulty in accessing healthcare facility; and the effects of general insecurity.

The baseline assessment in May 2014 and the midterm assessment in November 2015 were conducted in 317 randomly selected settlements at high risk for polio transmission. In each settlement, a total of 10 households were randomly selected, and all children aged <5 years were eligible as the study population. The total population of children in this age group sampled in 2014 was 3873, compared with 4651 in 2015. As revealed through an independent sample *t* test, the variables of interest in these 2 samples did not differ significantly. For purposes of this study, unweighted averages will be used to report on the change in immunization cards seen as well as the full immunization status among children aged 12–23 months.

## RESULTS

A total of 407 staff are supporting provision of dedicated mobile services in 3346 settlements at high risk for polio transmission, located in 480 wards and 98 local government areas in the 6 states (Table 1). A mobile team is made up of a nurse/midwife, 2 community health extension workers, a vaccinator, and a data clerk. The teams make quarterly visits to approximately 40 sites each in 3 months. The quarterly visits resulted in sufficient contact to achieve herd immunity against polio. The outreach team members provide integrated PHC services, which include polio and RI services. The service data generated by the teams is captured into the routine health management information system through the nearest PHC facility.

**Table 1.** Local Government Areas (LGAs), Wards, and Settlements Covered by Dedicated Mobile Teams, by State, Nigeria 2014–2015^a^

State	Population in 2013	Population in 2015	LGAs, No.	Wards, No.	Settlements, No.	Mobile Outreach Team Members, No.
Bauchi	5 867 499	6 213 424	9	49	769	96
Borno	5 208 447	5 515 517	17	73	620	72
Kaduna	7 419 070	7 811 161	12	63	402	43
Kano	11 698 688	12 370 748	27	119	432	62
Katsina	7 084 003	7 458 385	21	109	562	59
Yobe	2 931 470	3 108 687	12	67	561	75
Total	40 209 177	42 477 922	98	480	3346	407

^a^ Source: United Nations Children's Fund (UNICEF) and World Health Organization project reports, 2016.

**Figure 1.**
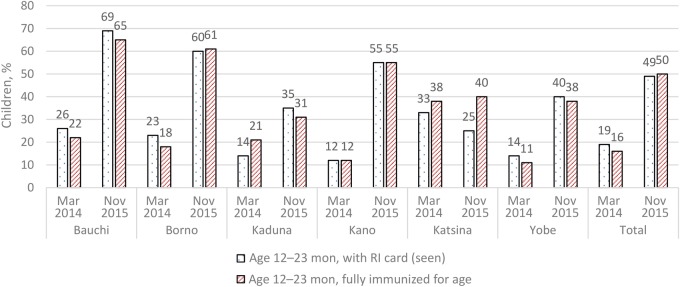
Percentage of children aged 12–23 months in the settlements at high risk for polio transmission seen with a routine immunization (RI) card and fully immunized for age at the start of the outreach program in March (Mar) 2014, compared with data generated in November (Nov) 2015. Source: Draft midterm evaluation of the project “Reaching Underserved and Isolated Communities in Northern Nigeria, 2016” (unpublished).

We reviewed data for the percentage of children aged 12–23 months in the settlements at high risk for polio transmission with an RI card seen and fully immunized for age at the start of the outreach program in March 2014 and compared with data generated during the midterm assessment in November 2015 for the 6 states that commenced implementation of activities in 2014 (Bauchi, Borno, Kaduna, Kano, Katsina, and Yobe) (Figure 1). The data showed an overall average increase in the percentage of children aged 12–23 months in the settlements at high risk for polio transmission with a RI card seen, from 23% (234 of 1017; median, 23%; quartile 1, 16%; quartile 3, 27%) to 56% (710 of 1269; median, 59%; quartile 1, 39%; quartile 3, 63%) and an overall average increase in fully immunized children aged 12–23 months, from 19% (194 of 1017; median, 17%; quartile 1, 15%; quartile 3, 23%) to 55% (695 of 1269; median, 52%; quartile 1, 41%; quartile 3, 64%) (Figure 1). The increase in service delivery figures represents the contribution of the dedicated mobile teams and polio VCMs to the delivery of RI services.

Starting in 2013, resources from the polio eradication program were used to set up a network of VCMs in high-risk settlements in 12 states of Northern Nigeria at high risk of polio transmission. For purposes of this study, the services generated by the polio VCM network in the initial 6 states (Bauchi, Borno, Kaduna, Kano, Katsina, and Yobe) will be examined. The polio eradication program also involved traditional and religious leaders at all levels in a bid to minimize resistance to the poliovirus vaccine and mobilize communities for the services. The engagement helped diffuse the hostile environment for polio eradication in the face of rejection and insecurity based on then-prevailing cultural norms and religious practices. The strategy of involving community, religious leaders, and household heads was also used in the selection of VCMs in the communities.

A VCM was identified from each of the settlements at high risk for polio transmission and trained on how to conduct household microcensus, home visits, administration of oral OPV, and tracking and linking of newborns with routine services in the nearby health facilities. Both the number of newborns reached with OPV0 (first dose of OPV according to the RI schedule) and zero-dose OPV (the first OPV dose for children aged >12 months who were not vaccinated according to the RI schedule) significantly increased between 2013 and 2015 (Table 2). The largest increase for OPV0 was seen in 2014, whereas zero-dose OPV showed a gradual increase.

**Table 2.** VCMs and Newborns Given OPV0 or Zero-Dose OPV With the Assistance of VCMs, by State, Nigeria 2013–2015^a^

State	VCMs, No.	Newborns Given OPV0 in the State With Assistance of VCMs, No.			Newborns Given Zero-Dose OPV With Assistance of VCMs, No.		
		Nov 2013	Dec 2014	Oct 2015	Nov 2013	Dec 2014	Oct 2015
Bauchi	450	120 488	167 508	173 225	770	6765	5880
Borno	746	64 734	114 882	77 269	1	10	2778
Kaduna	1613	163 716	226 613	204 854	2431	13 482	27 786
Kano	3808	181 654	355 774	267 148	7423	42 301	78 680
Katsina	2279	153 692	207 503	177 437	4807	27 721	39 154
Yobe	300	28 867	51 794	38 770	109	3594	3946
Total	9196	713 151	1 124 074	938 703	15 541	93 873	158 224

Abbreviations: OPV, oral poliovirus vaccine; OPV0, first dose of OPV according to the routine immunization schedule; VCMs, volunteer community mobilizers.

^a^ Source: United Nations Children's Fund (UNICEF) VCM database.

**Figure 2.**
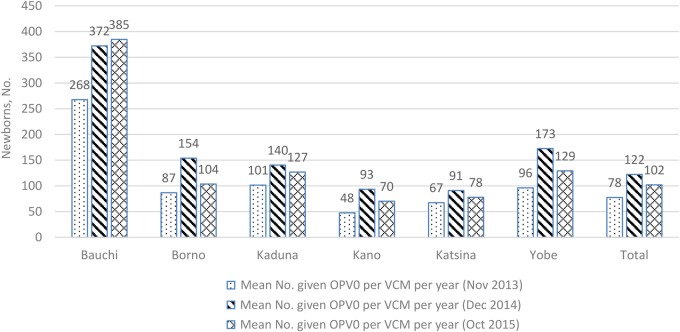
The mean number of Nigerian newborns receiving oral poliovirus vaccine (OPV) per volunteer community mobilizer (VCM) by reporting period and state. Source: United Nations Children's Fund (UNICEF) VCM database. Abbreviation: OPV0, first dose of OPV according to the routine immunization schedule.

Following a similar trend, the mean number of infants aged <12 months receiving OPV per VCM by reporting period showed a general increase from 2013 to 2015. Most of the increase occurred in 2014, with a general slight reduction in 2015 (Figure 2). The slight reduction may reflect 2 months of missing data in 2015. Even so, the mean number of children provided with OPV0 per VCM per month increased from 8 in 2013 to 11 in both 2014 and 2015.

**Figure 3.**
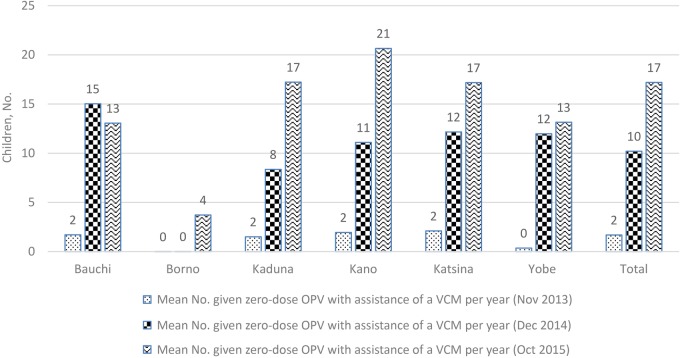
The mean number of Nigerian children receiving zero-dose oral poliovirus vaccine (OPV) per volunteer community mobilizer (VCM) by reporting period and state. Source: United Nations Children's Fund (UNICEF) VCM database.

The mean number of children aged >12 months receiving zero-dose OPV per VCM by reporting period showed a general increase from 2013 to 2015. Most of the increase occurred between 2013 and 2014 (Figure 3). The VCMs in the settlement conducted home visits and tracked 167 092 newborns in 2015, compared with 103 700 in 2014, and they also linked 156 537 infants aged <1 year to RI in 2015, compared with 95 550 in 2014 (Table 3).

**Table 3.** VCMs, Newborns Tracked by VCMs, and Infants Aged <1 y Linked by VCMs to Facility for RI, by State, Nigeria 2013–2015^a^

State	VCMs, No.	Newborns Tracked by VCMs, No.			Infants Aged <1 y Linked by VCMs to Facility for RI, No.		
		2013	2014	2015	2013	2014	2015
Bauchi	450	925	7442	5964	773	6964	5956
Borno	746	1	16	2913	1	6	1
Kaduna	1613	3368	16 669	30 456	2864	13 608	28 439
Kano	3808	8382	44 249	81 480	7948	42 985	78 933
Katsina	2279	6307	30 951	41 754	5034	28 501	39 614
Yobe	300	116	4373	4525	96	3486	3594
Total	9196	19 099	103 700	167 092	16 716	95 550	156 537

Abbreviations: RI, routine immunization; VCMs, volunteer community mobilizers.

^a^ Source: United Nations Children's Fund (UNICEF) VCM database.

## DISCUSSION

As in other countries, the network of human resources engaged for polio eradication in Nigeria is large [12–15]. During the initial years of PEI, the contribution of the eradication efforts toward other health outcomes was little understood and largely unexploited [12, 13]. In later years, however, greater interest in and exploitation of the polio assets for other health outcomes started emerging in the literature. The quality and frequency of immunization activities combined to help prevent poliovirus transmission in the high-risk area of Northwest Nigeria, and the scaling up of RI for undervaccinated populations demonstrated the potential to increase population immunity, hence decreasing the need for SIAs [16]. Other related research findings also support the view that increased RI coverage reduces the incidence of polio and makes eradication feasible [17]. The targeting of high-risk areas and populations increased poliovirus vaccination coverage in Ethiopia, India, and Angola [18].

In the case of Nigeria, some of the existing polio personnel are used to increase access to poliovirus vaccination, RI, and selected high-impact PHC services in particular settlements at high risk for polio transmission. The main service package is made up of RI for the infants aged <1 year, and supplementary immunization for other children aged <5 years, including poliovirus vaccinations. The other interventions provided to this age population include vitamin A, deworming, screening for malnutrition, treatment of diarrhea with zinc and low-osmolality oral rehydration salts, treatment of malaria and pneumonia, and treatment of other minor ailments. Pregnant women are provided with antenatal care services, including tetanus toxoid vaccination, intermittent preventive therapy for malaria, iron and folate, and the promotion of key household practices. It has been observed that better synergy with PEI can increase vitamin A coverage, encourage the provision of better laboratory services, and improve community linkages [13].

This article offers evidence on the use of polio personnel to increase immunization coverage, delivery of other basic PHC services, and improved community linkages. Over a period of 21 months, the percentage of children aged 12–23 months seen with a RI card increased from a mere 19% to 49%. Full immunization coverage went up by a similar margin during the same period, from 16% to 50%. The increase in routine coverage was significant but still lower than the expected coverage of >80%, an indication that more efforts are needed to cover the gap. In addition, the performance of the dedicated mobile teams and polio VCMs should be analyzed to identify bottlenecks preventing the program from achieving the desired level of coverage.

The selected settlements at high risk for polio transmission were also provided with a community-based services delivery platform used for community social mobilization and demand generation. The network of 9196 VCMs in the 6 states delivered increasing OPV0 doses, from a mean of 78 per VCM in 2013 to 122 in 2014 and 102 in 2015. The mean number of children receiving zero-dose OPV per VCM increased from 2 in 2013 to 17 in 2015. The use of polio personnel reached newborns in the settlements at high risk for polio transmission in ways that have previously not been demonstrated. The VCMs conduct routine home visits, with particular emphasis on homes with pregnant women or newly delivered newborns. The provision of OPV0 to newborns complements the provision of RI services by the mobile teams that reach each of the settlements on a quarterly basis, whereas the zero-dose OPV complements the SIAs. The linking of newborns to facilities for RI is a demand generation intervention for RI and other newborn-related PHC services.

The range of services beyond poliovirus vaccination provided using polio resources reinforces the integration and strengthening of PHC as a sustainable delivery mechanism for RI and other related PHC services. That it is possible to provide these services in the settlements at high risk for polio transmission is evidence that targeting these settlements is possible, assuming an integrated approach for increased access to services works. The analysis of the use of polio personnel for RI demonstrates the existing use of the resources, including the gains that are being made. The analysis also highlights opportunities that could be used to increase targeting and provision of integrated services. The VCM structure needs to be considered for scale-up as part of PHC approach in hard-to-reach communities, especially because of its potential to reach newborns in a country with a slowly declining mortality rate in children aged <5 years and stagnated progress with neonatal mortality rates. Given that Nigeria still has to focus on polio eradication after reporting 2 new cases in August 2016, the dedicated mobile teams and VCMs will continue with their focus on polio eradication.

Our study had some limitations. Its findings are based on data collected in settlements at high risk of polio transmission, different from the average settlements in the respective states. Given the distinct nature of the settlements, the study findings may not be generalizable for the entire state. Furthermore, the dedicated mobile teams and the VCMs are not part of the normal health system structure, so their performance as assessed in the study does not reflect the performance of the entire health system and should therefore not be interpreted as such. This study sourced some data from government and United Nations agencies documents. The quality of these data sources may not be guaranteed, because these sources are not always subjected to rigorous quality assurance.
